# A psychosocial network approach studying biomedical HIV prevention uptake between 2017 and 2019

**DOI:** 10.1038/s41598-023-42762-2

**Published:** 2023-09-27

**Authors:** Hanne M. L. Zimmermann, Udi Davidovich, Ward P. H. van Bilsen, Liza Coyer, Amy Matser, Maria Prins, Frenk van Harreveld

**Affiliations:** 1 Department of Infectious Diseases, Public Health Service of Amsterdam, Nieuwe Achtergracht 100, 1018 WT Amsterdam, The Netherlands; 2 Department of Work and Social Psychology, Maastricht University, Maastricht, The Netherlands; 3 Department of Social Psychology, University of Amsterdam, Amsterdam, The Netherlands; 4 Department of Internal Medicine, Amsterdam Institute for Infection and Immunity Institute (AII), Amsterdam UMC, Academic Medical Center, Amsterdam, The Netherlands; 5 National Institute for Public Health and the Environment (RIVM), Bilthoven, The Netherlands

**Keywords:** Human behaviour, Epidemiology

## Abstract

Biomedical HIV-prevention strategies (BmPS) among men who have sex with men (MSM), such as pre-exposure prophylaxis (PrEP) and viral load sorting (VLS), are essential but relatively new and their uptake gradual. Using an extension of the causal attitude network approach, we investigated which beliefs are related to uptake of PrEP and VLS at each time-point. We included 632 HIV-negative MSM from the Amsterdam Cohort Studies from four data-waves between 2017 and 2019. We estimated weighted, undirected networks for each time-point, where we included pairwise interactions of PrEP and VLS uptake and related beliefs. PrEP use increased from 10 to 31% (*p* < 0.001), while VLS was reported by 7–10% at each time-point. Uptake of both BmPS was directly related to the perceived positive impact of the strategy on one’s quality of sex life and perceived supportive social norms. Overall network structure differed between time points, specifically in regard to PrEP. At earlier time points, perceptions of efficacy and affordability played an important role for PrEP uptake, while more recently social and health-related concerns became increasingly important.The network structure differed across data-waves, suggesting specific time changes in uptake motives. These findings may be used in communication to increase prevention uptake.

## Introduction

In most high-income countries, men who have sex with men (MSM) continue to be disproportionally affected by HIV^1^. In 2020, 63% of new HIV diagnoses in the Netherlands were among MSM^2^. In the early stages of the HIV epidemic, HIV prevention choices were limited to condom use, serosorting, or sexual abstinence. In the past decade, the HIV prevention toolbox has expanded with biomedical prevention strategies (BmPS), which make use of antiretroviral medication to prevent HIV infections. Two BmPS that effectively prevent HIV are pre-exposure prophylaxis (PrEP) and increased testing and treating of HIV-positive individuals (i.e., “treatment as prevention” [TasP]). PrEP is a pill containing antiretrovirals that can be taken either daily or intermittently (i.e., before and after sex) by HIV-negative individuals. If PrEP is taken correctly, it is highly effective in preventing HIV infection among MSM^3–6^. TasP enables HIV-negative MSM to decide to have safe sex with HIV-positive MSM based on information about their viral load, which we refer to as viral load sorting (VLS). VLS is based on the assumption of “Undetectable = Untransmittable” (U = U), which holds that HIV-positive individuals who have a sustained undetectable viral load—usually achieved by consistently adhering to antiretroviral therapy—cannot sexually transmit HIV^7,8^.

Mathematical modeling studies show that BmPS have the potential to eliminate new HIV infections among MSM, although this is highly dependent on BmPS uptake and adherence ^9–11^. Literature illustrates that BmPS uptake is often far from optimal^12–15^ with several psychosocial factors curtailing uptake^16–23^. An estimate of PrEP uptake suggests that only 7% of MSM in Amsterdam, the capital of the Netherlands, used PrEP in 2017^12^. At that time, PrEP was only available free-of-charge through research programs for a small number of MSM^24,25^. Outside research settings, PrEP was also used, but difficult to obtain and expensive, limiting its uptake^26,27^. In recent years, PrEP became more affordable and accessible due to the availability of generic PrEP in 2018^28^ and the implementation of a national PrEP program in August 2019^29^. PrEP uptake is currently estimated at 7542 individuals in the Netherlands^15,30^, which still falls behind the optimal uptake of 10,000 individuals who are eligible for PrEP^31^. As for VLS, no data is currently available on the number and proportion of HIV-negative MSM in the Netherlands that apply VLS as an active prevention strategy, despite the recent conclusive evidence for the effectiveness of U = U in the context of anal sex among MSM^8,32^. To optimize uptake of BmPS among those with unmet prevention needs, it is essential to get insight into actual uptake among MSM, to identify individual motives and barriers for BmPS usage and to explore how these change over time due to increased availability and acceptability within the MSM community. The present research aims to provide such insight.

To understand and explain the uptake of health behaviors, several psychosocial theories, which include the health belief model (HBM)^33^ and the theory of planned behavior (TPB)^34^, have successfully been used in the past. In the HBM, behavior is explained by the perceived threat of a health risk and individual motivation based on the perceived costs and benefits of a health behavior, self-efficacy and cues to action. In the TPB, behavior is explained through behavioral intention, which is the product of attitude, perceived behavioral control and social norms. In the context of PrEP, previous studies have shown barriers of PrEP uptake that were in line with such models, such as perceived lack of self-efficacy, low HIV risk perception, and problematic beliefs regarding PrEP’s efficacy, costs, expected stigma, and knowledge regarding the potential physical health and sexual effects^17,19–22^. The little that is known on the psychosocial factors related to application of VLS among HIV-negative MSM suggests that lack of knowledge and disbelief in the U = U principle are predominant factors of perceived low efficacy of VLS^35^. However, the fact that these models offer a simplified and causal explanation of behavior has been criticized as it assumes unidirectional effects of the predictors on behavior, which may mask important interrelationships between variables^36–38^. Furthermore, not all aspects of these models may be equally important, but instead there may be more central and less central components determining BmPS uptake. In addition, the adaptation of new preventive measures based on new scientific insights can be expected to change rapidly over time after which initial acceptability barriers give place to more structural ones. Such temporal changes have not received sufficient attention in previous studies on BmPS uptake.

Given the complexity of the psychological factors underlying BmPS uptake, we aim to examine more comprehensive interactions of factors that contribute to the uptake of BmPS over time and employ an approach that is agnostic about the causal relationships between factors. Using the causal attitude network (CAN) approach^39^, we applied a complex system network approach to provide insight into the relative importance of each specific factor that may be useful to target in behavior change interventions to improve the uptake of BmPS among MSM.

### Psychological network approach

**Figure 1:**
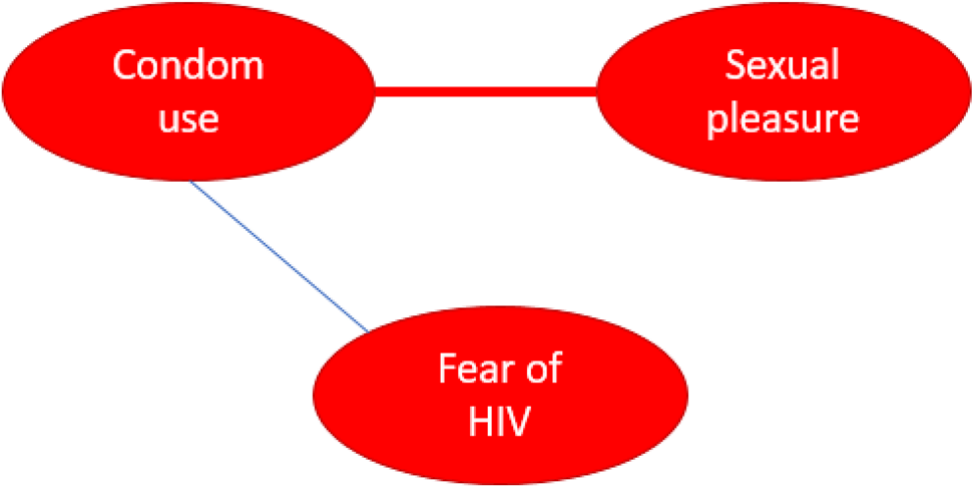
Example of a simple undirected weighted network with 3 nodes (represented by circles) and 2 edges (represented by lines). The magnitude of the connection is indicated by edge width. A blue line represents a positive and a red line represents a negative association.

In this study, we build on the work of Dalege and colleagues^38^ who introduced the Causal Attitude Network (CAN) model. Within this model, attitudes are conceptualized as a network of evaluative reactions (nodes) that interact with each other (edges) and are corrected for all other nodes in the network. The CAN model provides a novel approach to the study of attitudes and behaviors. This approach is inherently flexible and can encompass a wide range of factors that can include or exclude the target behavior. Including the target behavior in the network allows the exploration of direct and indirect relationships between specific beliefs and the target behavior^40^. An example of a hypothetical simple, weighted, undirected network in which the outcome of interest is included is presented in Fig. 1 below, in which three nodes are included: the behavioral HIV prevention strategy condom use and two evaluative reactions (fear of HIV and sexual pleasure). The connections between them are represented by edges that represent positive or negative relations between nodes and that can differ in magnitude (i.e., weighted network). The edges are undirected as they present bidirectional associations. Because of interrelatedness of nodes in the CAN model, influencing one node (e.g., sexual pleasure) may have consequences for other nodes within the network and result in change in the target behavior (i.e., condom use), or vice versa.

Other key properties of an empirical attitude network model include the overall *connectivity* of a network^41^, *centrality of nodes* within the network, and closely connected nodes that form *communities*. *Connectivity* is the extent to which the nodes in the network are related to one another. It has been shown that the higher the connectivity of the network, the more closely its components are related to behaviour and the more stable and resistant to change or persuasion the network is^38,42^. The *centrality* of a node reflects the extent to which it is connected to other nodes in the network. Centrality thus provides information about the structural importance of a given node in the network and may inform which nodes are therefore most closely related to decision-making^39,42^. Beliefs clustering with the target behaviour (i.e., *community*) indicate they are more closely connected to each other than to other nodes. In sum, all network properties provide some information on which nodes are most important within the network, but also provide information on their mutual dependencies with other nodes.

### The present study

So far, the CAN model has only been applied to cross-sectional attitude data towards a single attitude object. Temporal network approaches to date have been theoretical and predominantly focused on the connectivity property of network models^41,43^. This is one of the first study in which the interplay of factors is investigated through empirical data. We explore which factors relate to two co-existing BmPS and how changes in these factors may help explain how the uptake of PrEP and VLS change across time points. We extend the CAN model in two important ways. First, we extend the model with factors that transcend latent constructs of individual attitudes. We investigate a broader psychological system that also includes factors related to perceived structural and practical barriers of BmPS uptake, knowledge and demographics. Second, we focus on several properties of the CAN model at different time points which helps us to evaluate whether the structure of the network and thus the relative importance of factors is different at different time points. In doing so, our study is not only the first to apply such a complex attitude network approach to two recently introduced BmPS, but also one of the first to examine the temporal dynamics of attitude networks.

## Results

**Figure 2:**
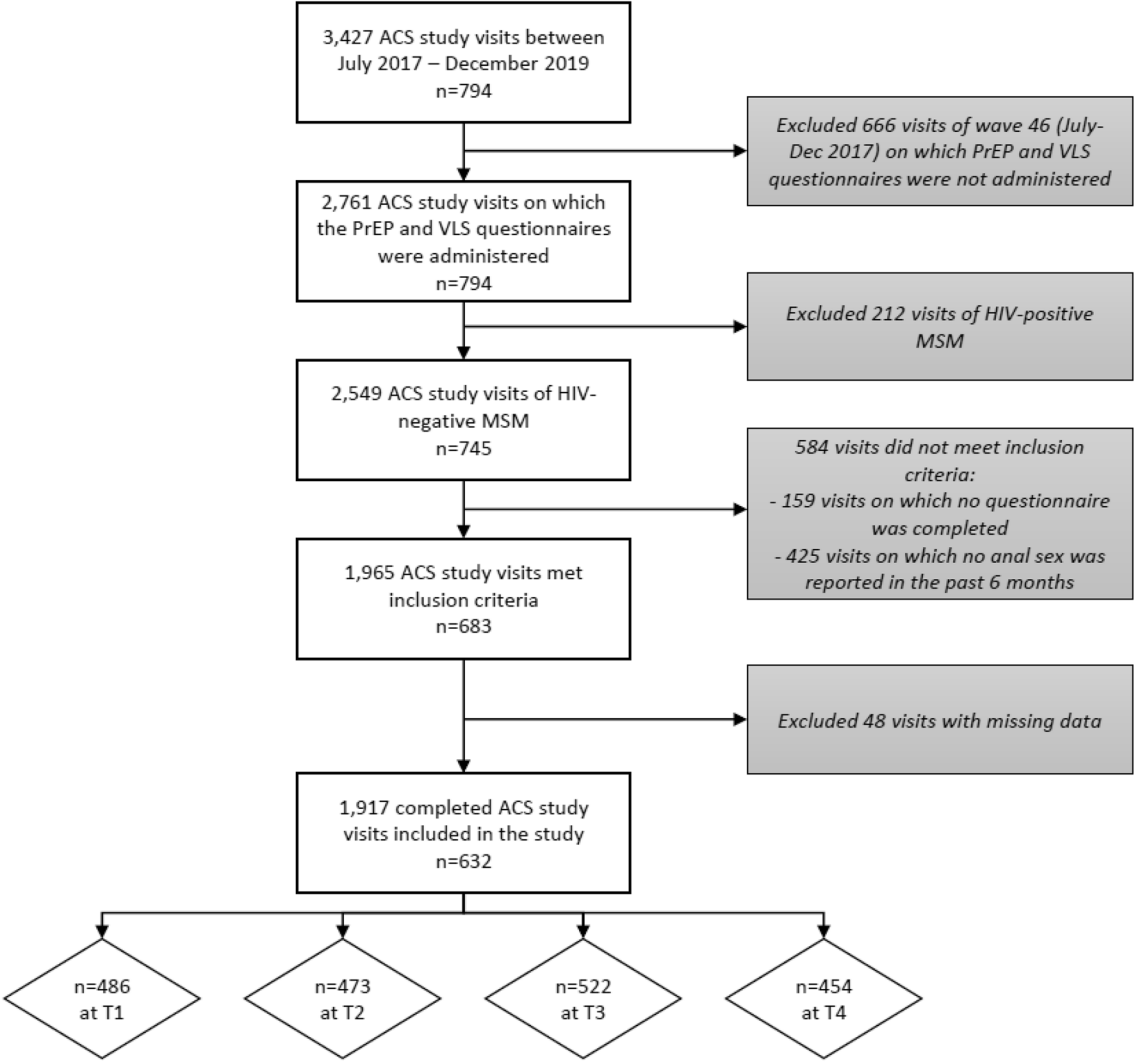
Flowchart of the included ACS study participants per time point. *ACS* Amsterdam Cohort Study on HIV, *PrEP* pre-exposure prophylaxis, *VLS* viral load sorting, *HIV* Human Immunodeficiency Virus, *MSM* men who have sex with men.

In total, we included 632 HIV-negative MSM (Fig. 2) with a median of 3 [interquartile range (IQR)]: 2–4) visits (see Table 1 for their baseline socio-demographic characteristics). Use of any HIV prevention strategy in the past 6 months increased from 78% at T1 to 84% at T4 (test for linear trend: odds ratio (OR) = 1.10, 95% confidence interval (95% CI) = 1.03–1.17; *p* = 0.003, Table 2). PrEP use increased from 10% at T1 to 31% at T4 (test for linear trend: OR = 1.40, 95% CI 1.31–1.49; *p* < 0.001), condom use decreased from 64 to 57% (test for linear trend: OR = 0.92, 95% CI 0.88–0.97; *p* = 0.002), and uptake of VLS remained similar and was overall reported in 7–10% of participants (test for linear trend: 1.06, 95% CI 0.95–1.18; *p* = 0.278).

**Table 1:** Socio-demographic and study characteristics of HIV-negative MSM of the Amsterdam Cohort Studies, July 2017–December 2019, Amsterdam, the Netherlands.

Total number of participants	632
Total number of visits	1917
Number of visits per participant (median, IQR)	3 (2–4)
1 visit	54 (8%)
2 visits	130 (21%)
3 visits	189 (30%)
4 visits	259 (41%)
Age at baseline^a^ (median, IQR)	41 (31–49)
Born in the Netherlands	527 (83%)
Living in Amsterdam^a^	523 (83%)
Exclusively homosexual^a^	509 (81%)
College degree or higher^a^	504 (80%)
Having a steady partner^b^	408 (65%)
> 5 casual partners	326 (52%)

*MSM* men who have sex with men, *HIV* human immunodeficiency virus.

^a^At inclusion in Amsterdam Cohort Studies on HIV.

^b^At baseline, defined as the first visit of the participant between 1 July 2017 and 31 December 2019.

**Table 2:** Use of HIV prevention strategies in the past 6 months per time point and time trends among 632 HIV-negative MSM within the Amsterdam Cohort Studies, July 2017–December 2019, Amsterdam, the Netherlands.

	T1 (N = 468)		T2 (N = 473)		T3 (N = 522)		T4 (N = 454)		OR (95% CI)	*p*-value^a^
	n	%	N		n	%	n	%		
Any HIV prevention strategy^b^	363	78	356	75	408	78	381	84	1.10 (1.03–1.17)	0.003
Condom use during sex with a casual partner	301	64	250	53	279	56	254	57	0.92 (0.88–0.97)	0.002
PrEP	48	10	104	22	128	25	141	31	1.40 (1.31–1.49)	< 0.001
VLS	32	7	43	9	39	8	43	10	1.06 (0.95–1.18)	0.278
No HIV prevention	105	22	117	25	114	22	73	16	0.91 (0.85–0.97)	0.003
Any biomedical HIV prevention strategy^c^	72	15	127	27	151	29	158	35	1.31 (1.23–1.39)	< 0.001
Only PrEP, not VLS	40	9	84	18	112	21	115	25	1.39 (1.30–1.50)	< 0.001
Only VLS, not PrEP	24	5	23	5	23	4	17	4	0.93 (0.81–1.05)	0.239
Both PrEP and VLS	8	2	20	5	16	4	26	8	1.39 (1.23–1.58)	< 0.001
No biomedical HIV prevention	396	85	346	73	371	71	296	65	0.76 (0.72–0.81)	< 0.001

*MSM* men who have sex with men, *OR* odds ratio, *95%* *CI* 95% confidence interval, *PrEP* pre-exposure prophylaxis; *HIV* Human Immunodeficiency Virus, *VLS* viral load sorting.

^a^Test for linear trend examined in logistic regression models using generalized estimating equations to account for repeated measurements within individuals. The OR indicates the increase in the odds of the outcome with each subsequent time-point.

^b^Includes PrEP use, viral load sorting, condom use and any combination of these strategies.

^c^Includes PrEP use and viral load sorting and any combination of these strategies.

The means and standard deviations of all included PrEP and VLS beliefs at the four time points are shown in Table 3.

**Table 3:** HIV risk perception and PrEP and VLS-beliefs per time point among men who have sex with men within the Amsterdam Cohort Studies, January 2017–December 2019, Amsterdam, the Netherlands.

	T1 (N = 468)		T2 (N = 473)		T3 (N = 522)		T4 (N = 454)		Beta (95% CI)	*p*-value^a^
	M	SD	M	SD	M	SD	M	SD		
HIV risk perception	1.9	0.87	1.8	0.86	1.8	0.82	1.7	0.78	− 0.04 (− 0.06–− 0.02)	< 0.001
PrEP beliefs										
Impact quality of sex life	4.6	1.57	4.6	1.76	4.7	1.67	4.8	1.67	0.06 (0.02–0.10)	0.003
Impact on serodiscordant couples	4.6	1.59	4.7	1.71	4.8	1.67	4.8	1.66	0.06 (0.02–0.10)	0.005
Solidarity towards HIV-positive individuals	4.3	1.56	4.4	1.58	4.5	1.61	4.5	1.67	0.05 (0.01–0.09)	0.010
Efficacy	4.8	1.51	5.0	1.58	5.1	1.53	5.3	1.52	0.12 (0.08–0.15)	< 0.001
Essential for high-risk	5.4	1.42	5.7	1.46	5.8	1.38	5.9	1.39	0.11 (0.08–0.14)	< 0.001
Redundant	3.4	1.65	3.4	1.69	3.4	1.76	3.4	1.65	0.01 (− 0.03–0.06)	0.548
Affordability	2.9	1.46	3.7	1.57	3.8	1.63	4.1	1.70	0.29 (0.25–0.34)	< 0.001
Resistance development HIV medication	4.1	1.20	4.2	1.24	4.3	1.24	4.3	1.27	0.06 (0.02–0.09)	0.001
Burden side-effects	4.1	1.26	4.3	1.36	4.3	1.37	4.4	1.43	0.11	< 0.001
Burden PrEP procedures	4.1	1.31	4.3	1.41	4.3	1.50	4.2	1.52	0.05 (0.02–0.09)	0.004
Impact on sex life	2.9	1.39	2.6	1.43	2.7	1.41	2.5	1.37	− 0.08 (− 0.11–− 0.04)	< 0.001
Opinion relevant others PrEP use for HIV prevention	4.5	1.49	4.7	1.52	4.7	1.58	4.7	1.53	0.06 (0.02–0.09)	0.006
Gay friends use PrEP	3.1	1.52	3.6	1.61	3.7	1.77	3.9	1.78	0.21 (0.16–0.25)	< 0.001
Opinion gay friends PrEP use	4.8	1.32	5.1	1.27	5.0	1.40	5.1	1.41	0.08 (0.05–0.11)	< 0.001
Association increased sexual risk taking	3.5	1.73	4.7	1.57	4.6	1.55	4.6	1.59	0.27 (0.22–0.32)	< 0.001
Association sexual health responsibility	5.5	1.34	5.2	1.31	5.2	1.34	5.3	1.27	− 0.08 (− 0.11–− 0.04)	< 0.001
Association better sex life	4.5	1.48	4.1	1.57	4.2	1.46	4.3	1.48	− 0.04 (− 0.08–0.00)	0.065
Association promiscuity	4.0	1.62	4.4	1.55	4.4	1.53	4.4	1.54	0.10 (0.05–0.15)	< 0.001
Easier to use than condoms	4.4	1.54	4.8	1.74	4.7	1.68	4.8	1.68	0.09 (0.05–0.14)	0.002
Self-efficacy daily PrEP	4.4	1.37	4.8	1.49	4.7	1.45	4.9	1.45	0.13 (0.10–0.16)	< 0.001
Self-efficacy event-driven PrEP	4.3	1.22	4.7	1.31	4.7	1.30	4.7	1.26	0.11 (0.77–1.40)	< 0.001
Viral load sorting beliefs										
Prevents HIV transmission	4.6	2.01	5.2	1.95	5.0	1.88	5.0	1.82	0.11 (0.06–0.16)	< 0.001
Protects serodiscordant couples	4.8	1.68	4.8	1.73	5.0	1.59	4.9	1.59	0.03 (− 0.01–0.08)	0.139
Efficacy	3.9	1.82	3.9	1.74	4.1	1.77	4.0	1.83	0.02 (− 0.03–0.07)	0.407
Easier to use than condoms	3.5	1.74	3.3	1.77	3.4	1.80	3.4	1.79	− 0.03 (− 0.07–0.02)	0.202
Impact quality sex life	3.8	1.75	3.5	1.69	3.6	1.69	3.7	1.77	− 0.01 (− 0.06–0.03)	0.536
Opinion others to use viral load sorting for HIV prevention	3.2	1.47	3.1	1.40	3.2	1.44	3.2	1.40	− 0.01 (− 0.05–0.03)	0.552
Gay friends use viral load sorting	2.7	1.47	2.8	1.39	2.9	1.50	2.8	1.45	0.28 (− 0.01–0.07)	0.166
Opinion gay friends application of viral load sorting	2.9	1.49	3.1	1.44	3.2	1.50	3.2	1.43	0.08 (0.04–0.11)	< 0.001
Self-efficacy	3.7	1.47	3.4	1.47	3.5	1.53	3.5	1.52	− 0.04 (− 0.08–0.00)	0.038

*PrEP* pre-exposure prophylaxis, *VLS* viral load sorting; *95% CI* 95% confidence interval, *HIV* Human Immunodeficiency Virus.

^a^Test for linear trend examined in linear regression models using generalized estimating equations to account for clustering within individuals. The beta indicates the increase or decrease in the outcome with each subsequent time-point.

### Network analyses

**Figure 3:**
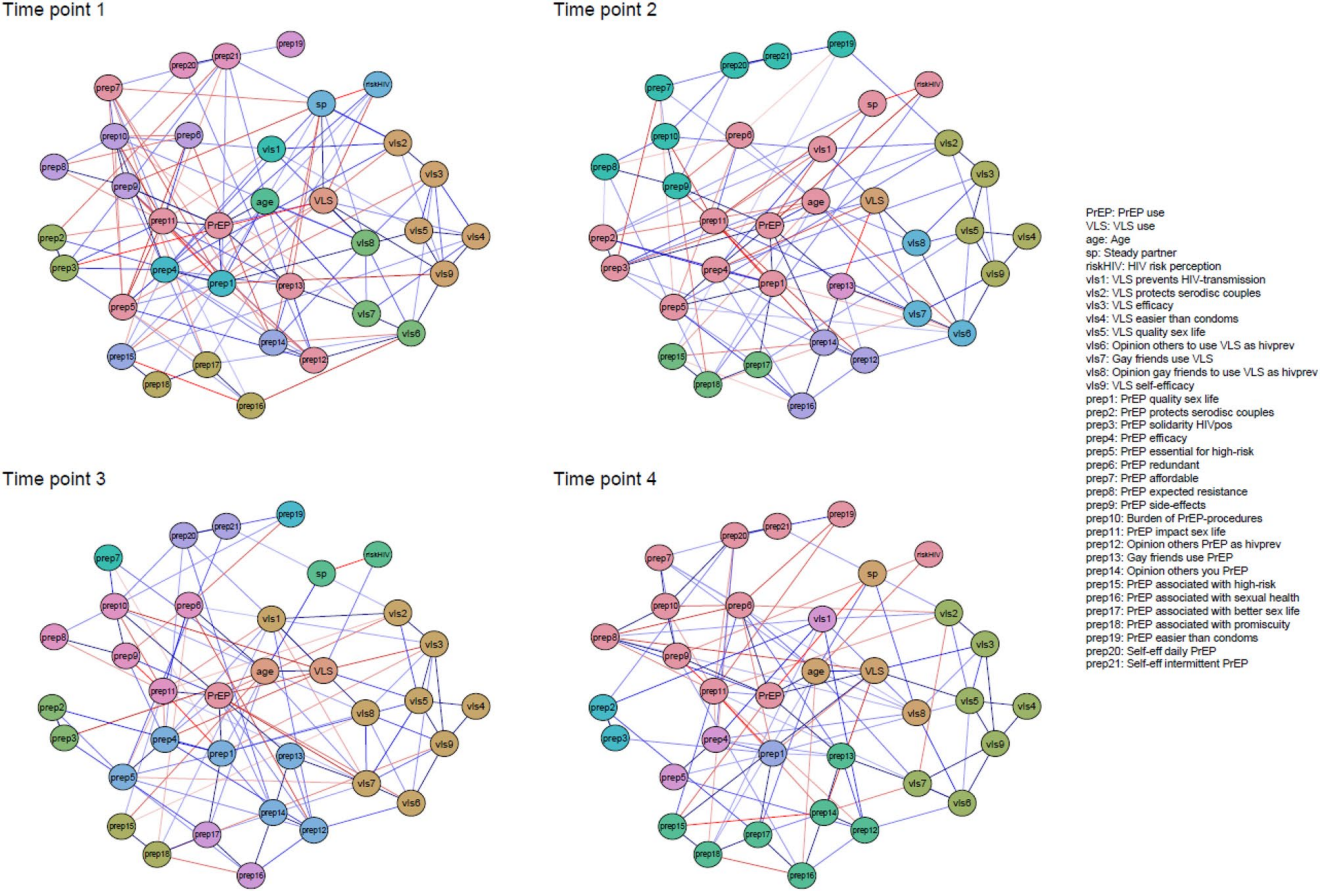
Estimated networks of PrEP use, VLS and related factors at each time point. Nodes represent the measured factors and edges represent the bidirectional relations. Positive relations are displayed with blue edges and negative associations with red edges. Stronger relations are displayed with thicker edges and greater color intensity. Edge weights below. 15 are omitted to increase clarity. For the relations between continuous nodes, edges can be interpreted as partial correlations. For relations with the binary nodes (PrEP, VLS and sp), a positive relation indicates that increasing the node results in a higher probability of outcome 1 of the factor (PrEP = 1: used PrEP in past 6 months, VLS = 1: reported VLS in past 6 months; sp = 1: reported a steady partner in the past 6 months). The colored groups indicated clusters of higher interconnectedness (i.e., *communities*). *PrEP* pre-exposure prophylaxis, *VLS* viral load sorting, *HIV* Human Immunodeficiency Virus.

The estimated networks, including use of PrEP and VLS as BmPS and related factors, are shown in Fig. 3 per time point. The edge weights, accuracy and difference tests of the estimated edges, and community detection analyses at each time point can be found in the Supplementary materials S1, S2, S3 and S4.

### Direct correlates of PrEP and VLS uptake

From T1 to T4, PrEP use was consistently and directly connected at each time point to a positive descriptive norm of gay friends using PrEP [prep13; edge weights at T1: 0.34 (0.01–0.71), T2: 0.18 (0.02–0.39), T3: 0.30 (0.22–0.60); T4: 0.27 (0.00–0.39)] and expecting lower burden of expected side-effects [prep9; T1: 0.48 (0.33–0.72), T2: 0.20 (0.00–0.37), T3: 0.12 (0.00–0.29), T4: 0.34 (0.00–0.51)], and at three time points to the perceived positive impact of PrEP on one’s sex life [prep1; T1:0.14 (0.00–0.41), T2:0.32 (0.18–0.57), T3:0.21 (0.02–0.39); and prep11; T1: 0.26 (0.01–0.45), T3: 0.27, T4: 0.07 (0.00–0.26)], older age [age; T1: 0.26 (0.02–0.78), T2: 0.14 (0.00–0.38), T3: 0.20 (0.10–0.47)] and VLS uptake [VLS; T1:0.09 (0.00–0.78), T2:0.09 (0.00–0.57), T3: 0.38 (0.00–0.76)]. Correlates directly related to PrEP uptake at T4 but not at earlier time points were not expecting PrEP to cause HIV drug resistance [prep8; T4: 0.12 (0.00–0.32)) or burdensome PrEP procedures (prep10; T4: 0.34 (0.00–0.38)], and not having a steady partner [sp; T4: − 0.18 (− 0.22–0.00)]. In contrast, beliefs such as PrEP’s perceived affordability [prep7; T2: 0.11 (0.00–0.37)] and efficacy [prep4, T1: 0.08 (0.00–0.12), T2: 0.16 (0.00–0.36)] were directly related to PrEP uptake at earlier time points (T1 and/or T2), but not at more recent time points (T3 and/or T4).

VLS uptake was directly related at most time points to PrEP uptake [PrEP, T1: 0.09 (0.00–0.78), T2: 0.09 (0.00–0.57), T4: 0.38 (0.00–0.55)], the perceived beneficial impact of VLS on quality of sex life [vls5; T3: 0.11 (0.00–0.31), T4: 0.24 (0.00–0.28)] and to gay friends approving of VLS as an HIV prevention method [vls8; T1: 0.25 (0.00–0.71), T2: 0.23 (0.00–0.51)]. Additionally, the strongest direct correlates of VLS uptake uniquely for T4 were expecting PrEP to cause HIV drug resistance [prep8; T4: − 0.11 (− 0.36–0.00)], viewing PrEP users as individuals engaging in high-risk behaviors [prep15; T4: 0.21 (0.00–0.32)], a negative injunctive norm of others not approving of PrEP use [prep14; T4: − 0.13 (− 0.19–0.00)], the perceived good efficacy of VLS [vls3; T4: 0.14 (0.00–0.16] and older age [age; T4: 0.14 (0.00–0.37)].

### Community detection

The colored groups in Fig. 3 represent clusters with higher interconnectedness (i.e., *communities*). Community detection analysis revealed that PrEP uptake at T4 clustered with practical aspects, such as perceived burden of side-effects, burden of PrEP procedures, self-efficacy and affordability, while at T1 clustering was suggestive of more social concerns or need for social approval, indicating differential interconnectedness of nodes between time points. Community detection of VLS uptake was less distinctive and suggests a role for socio-demographics, such as increasing age, and relationship status being related to VLS uptake.

### Node centrality

**Figure 4:**
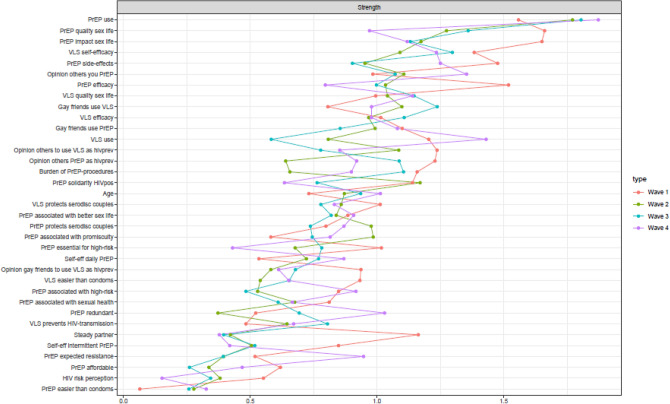
Centrality plot of the estimated networks of PrEP use, VLS and related factors at each time point. Strength indicates the influence of a node on the network as it is based on the sum of the absolute edge values that are connected to a node. A high score indicates that changing the specific node is more likely to have a profound effect on the network as a whole due to its relation with many other nodes. *VLS* viral load sorting, *PrEP* pre-exposure prophylaxis, *HIV* Human Immunodeficiency Virus.

The node centrality results are displayed in Fig. 4 and represent which nodes are most influential in the networks. Outcomes of the accuracy and differences tests in strength per time point can be found in the supplementary material S5 and S6. The average correlation of the strength of our original samples with the strength of subsets of that sample suggest that the strength measure was highly stable and accurate at all time points (all above 0.7). Across time points, the strength of our outcomes of interest, PrEP and VLS uptake, increased and became the nodes with highest strength at T4 (prep T4: 1.9; vls T4: 1.4) strength did not differ). Regarding PrEP beliefs, largest change in strength was found in PrEP’s perceived impact on one’s sex life and quality of sex life and perceived efficacy of PrEP. These nodes were most influential at T1 for the network (prep1 and prep11 T1 both 1.7; prep4 T1: 1.6), but their relative importance decreased towards T4 (prep1 T4: 0.9; prep11 T4: 1.2; prep4 T4: 0.8), showing a similar pattern to the direct correlates of PrEP use. In contrast, nodes with increasing strength from T1 to T4 were not expecting PrEP to cause HIV drug resistance (prep8 T1: 0.5; T4: 0.9) and others approving of PrEP use (prep14 T1: 1.0; T4: 1.3), which were also direct correlates of PrEP use at T4. Other PrEP beliefs showed relatively stable but lower strength, suggesting that influencing these nodes are less likely to change the network at T4. The strength of VLS beliefs were fairly similar for all VLS nodes and remained relatively similar across time points, although some VLS-related social norm nodes lost strength, suggesting a more important role in the network at T1 compared to T4. At T4, VLS nodes with the highest and similar strength were perceived self-efficacy of VLS use (vls9 T4: 1.5) and the perceived preventive effectiveness of VLS (vls3 T4: 1.0), and others using VLS (vls7 T4: 1.1) and approving of VLS as HIV prevention strategy (vls6 T4: 0.9).

### Temporal network differences

**Figure 5:**
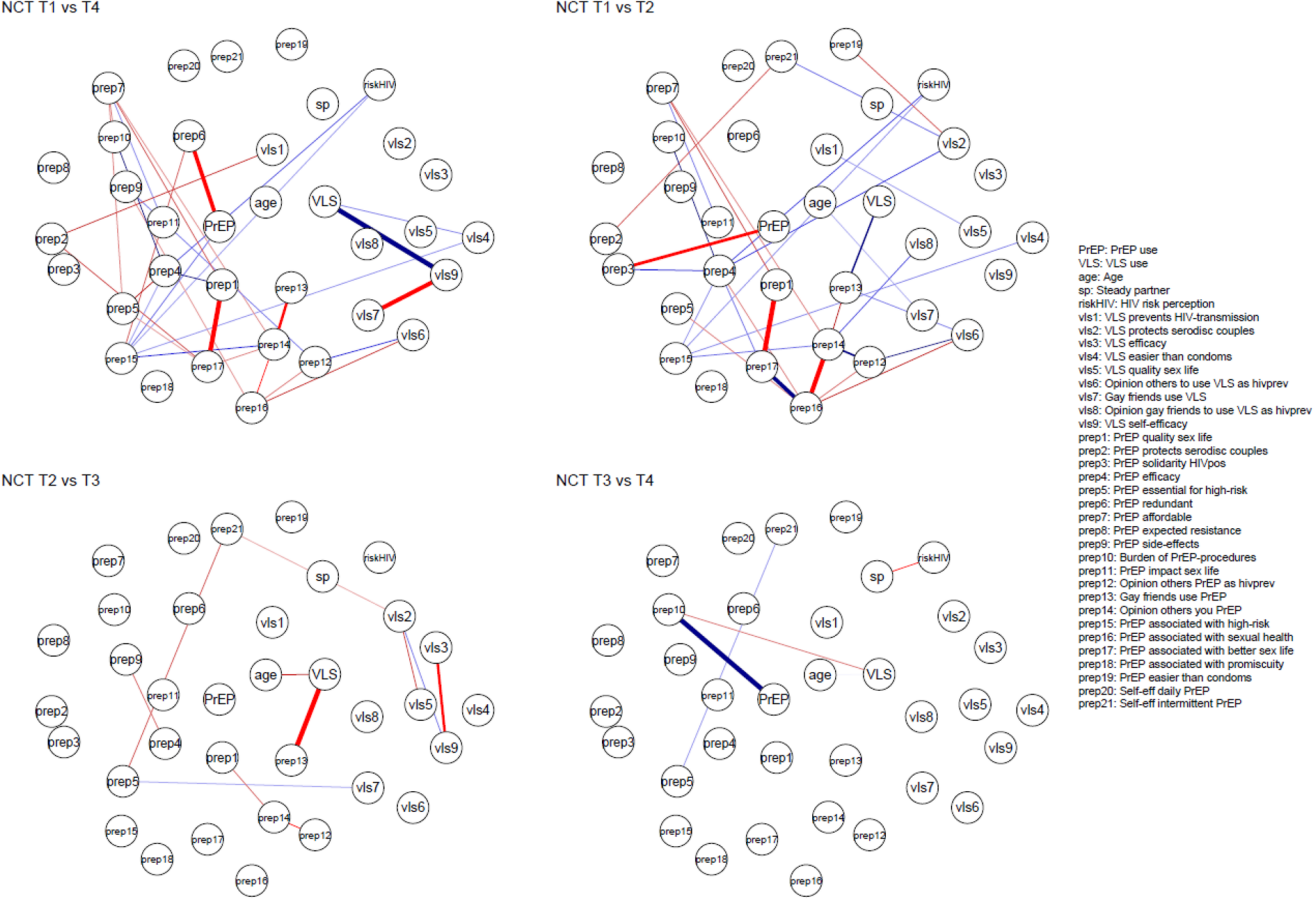
Significant differences in edge weights between time points (T1 vs. T4; T1 vs. T2; T2 vs. T3; T3 vs. T4) obtained from the network comparison test (NCT). The magnitude of the edge differences is indicated by edge width. A blue edge indicates that the relation (based on edge weights) is significantly weaker, absent or more negative at the later time point compared to the earlier time point. A red edge indicates that the relation is significantly weaker, absent or more negative at the earlier time point. Edge weights below. 15 are omitted to increase clarity. Note that the NCT compares networks of either continuous or binary variables, and not from mixed networks using mgm. The results of the NCT (i.e. significant differences between edges) are therefore based on the networks estimated for continuous variables (with EBICglasso). The NCT graph displays those edges that differ significantly according to the results of the NCT with the displayed difference in strength based on the edge weight in the mgm network. *VLS* viral load sorting, *PrEP* pre-exposure prophylaxis, *HIV* Human Immunodeficiency Virus.

The results of the network comparison test indicate that the global strength (i.e., a measure of the connectivity of the network) did not change across time points (global strength for T1: 10.75, T2: 9.92, T3: 11.83, T4: 10.69; *p* > 0.05 for all, Table 4). However, the network structure differed significantly between T1 and T4 (*p* = 0.020) and T1 and T2 (*p* = 0.002), suggesting that the overall difference between T1 and T4 is most likely due to changes in network structure occurring from T1 to T2. Figure 5 displays the significant differences in edge weights between nodes between each of the time points.

**Table 4:** Results of the network comparison test based on global strength and network invariance.

Networks		Global strength	Network structure invariance
Time point	Time point	*p*-value	*p*-value
T1	T4	0.967	0.020
T1	T2	0.487	0.002
T2	T3	0.147	0.642
T3	T4	0.386	0.863

### Sensitivity analyses

The sensitivity analyses in which we restricted our analyses to those reporting condomless anal sex (CAS) showed similar results with regards to baseline characteristics (Supplement S9), associations with PrEP and VLS uptake (Supplement S7 and S8), and the network structure (network invariance: *p* = 0.594) (Supplement S10 and edges directly relating to PrEP and VLS at T4 (Supplement S1). In addition, PrEP use but not VLS, also increased among both participants reporting and not reporting CAS with a casual partner (*p* < 0.001 for both, data not shown).

## Discussion

Since uptake of BmPS is one of the key factors to achieve HIV elimination, we describe the interplay of factors that were related to the uptake of these strategies among HIV-negative MSM and their related temporal dynamics. To do so, we took a complex systems approach by applying the CAN model, in which variables are presented as networks, to the case of BmPS uptake. We found that, between July 2017 and December 2019, PrEP use significantly increased, while the uptake of VLS remained relatively stable and was reported by approximately one tenth of participants. We showed that at almost all time points, both strategies were interrelated: the uptake of each strategy was directly related to the use of the other strategy. Other consistent relations of BmPS uptake were the perceived positive impact of each strategy on one’s quality of sex life and significant others approving or using of the strategy as an HIV prevention method. In addition, we found that the network structure significantly differed between the first and last time point, suggesting changes in uptake motives between time points, specifically regarding PrEP uptake.

Regarding these temporal changes, our study showed that perceptions of efficacy and affordability played a more important role in PrEP uptake at earlier time points as compared to later time points. This may in part be explained by the concurrent developments in PrEP implementation in the Netherlands increasing its affordability and accessibility since 2018. As shown by the difference in network structure between T1 and T2, this shift already occurred early in our study, after which the network structure remained stable until T4. We interpret this as an indication that once such initial concerns are overcome, social (e.g., others approval of PrEP use) and health-related barriers (e.g., not expecting side-effects, HIV drug resistance and burden of PrEP procedures) become more important in the decision-making process. In contrast to PrEP, increase in usability-related factors such as efficacy were not related to increased uptake of VLS. A possible explanation is that the conclusive scientific evidence for U = U in MSM only became available in mid-2018^32^ and therefore the trust in this strategy could have been low in the earlier data waves we used. Furthermore, as TasP is usually framed as an HIV-prevention strategy for HIV-positive individuals^44–46^ while our participants were all HIV-negative, they may not be aware of TasP as existing HIV prevention strategy in the form of VLS.

At the most recent time point, PrEP use and VLS became the most central nodes and showed the strongest direct positive relationships with each other. This is encouraging as it may imply that whenever one BmPS is used, the likelihood of using another BmPS is higher, suggesting that overall BmPS became more acceptable HIV prevention strategies to a subgroup of MSM. In addition, results of BmPS uptake and decision-making were comparable in sensitivity analyses restricted to participants reporting CAS. In line with this, previous studies have suggested that gaps between eligibility and uptake are more likely explained by psychological factors, such as those included in our study, than by behavioural factors^13,47^.

### Network methodology and its practical value for BmPS implementation

The added value of the CAN approach is that it is a data-driven approach to attitudes that provides insight into the structure of attitudes that goes beyond traditional models. It sheds light onto how the components that form a complex attitude system interrelate and adds a level of complexity to other models that describe unidirectional associations between a limited number of determinants and the behavioural outcome. In doing so, networks provide insight into how the factors are interrelated and show that not all factors—despite also being associated with BmPS uptake—are equally important at all time points.

A first key insight drawn from the present study is that it points towards specific perceptions that remain important across different time points and are consistently related to BmPS uptake, which include the impact of BmPS on the quality of sex life and social norms. Previous studies also found intimacy and increased sexual pleasure due to reduced need for condoms as important facilitators of PrEP uptake^49–53^. Perceived negative social norms were identified as a barrier for PrEP initiation, adherence and retention^20,54–57^. Our findings suggest that social norms and perceived quality of sex life remain closely linked to PrEP use and VLS, irrespective of stage of their implementation.

Second, the temporal changes in factors relating to PrEP uptake suggest that uptake motives may differ according to the stage of BmPS implementation. Given that new BmPS for HIV such as vaccines are currently being developed^58^ and new PrEP modalities such as long-acting injectable PrEP already showed efficacious^59^, this knowledge is helpful to decide which beliefs are most directly linked to BmPS uptake at different stages of implementation. In the future, such knowledge can be applied not only to new BmPS for HIV prevention that are still in development, but also to other fields that rely on biomedical prevention strategies, such as new vaccines or direct-acting antiviral medication for hepatitis C among MSM.

Third, previous studies have used the CAN network methodology to choose intervention targets. For example, earlier work indicated that influencing a node directly related to the target behaviour was a successful means to generate behavioural change in the target behaviour^48^. Another study showed that an intervention aimed at a central node led to change in the intended outcome and affected connected nodes^62^. In line with this reasoning, our results may also offer potential avenues towards behavioural change regarding BmPS uptake that are specific to the present context by looking at the nodes that either have the highest strength—and are thus highly influential in the network—or have direct associations with one or both BmPS at T4. This points towards the importance to target several aspects of social norms in the MSM community regarding BmPS, including gay friends and significant others approving of BmPS use and gay friends also applying these strategies. However, these nodes are likely more difficult to change than less influential nodes (e.g., lower in strength), but an effect of a change on the rest of the network is expected to be more profound and may result in more endurable behaviour change. Therefore, it may be more feasible to target nodes that are connected to these highly central nodes. In this case, *prep15* and *prep16* were directly connected with a relatively high effect size and describe beliefs linking PrEP to promiscuity or rather sexual health. A strategy to improve PrEP uptake might thus be to generate more positive community norms include further fortifying the framing of PrEP as a health preservation opportunity rather than a sign of promiscuity^20,63^. In addition, considering the undirectional nature of the network, it is also possible that PrEP use itself leads to supportive norms. PrEP-users themselves have indeed been described as role models who can facilitate peer communication and normalization of PrEP use^64,65^. In any case, both avenues underscore the added value of a network analysis in which the interrelatedness of factors is taken into account over other analysis techniques that only focus on associations with the outcome. It should be noted, however, that our findings only provided insights into potential routes for behaviour change based on the networks we presented at each wave. Future studies should focus on whether targeting the described intervention targets indeed results in change in uptake. Unfortunately, our results provided us less apparent directives how to intervene with VLS uptake in the current time due high interrelatedness of VLS uptake with PrEP use and socio-demographic factors (i.e., age and having a steady partner). The latter requires further investigation to understand their impact in VLS uptake. The current low uptake of VLS may suggest that much work can still be done to improve awareness of VLS as an effective HIV prevention strategy. Considering the strong relationship between beliefs in PrEP efficacy and PrEP uptake, increasing the recognition of VLS as an effective prevention strategy among HIV-negative MSM could further contribute to increased uptake. In turn, increased uptake could also lead to increased trust in effectiveness.

### Theoretical implications

Apart from the implications applied to HIV prevention, our study showed the viability of (1) broadening the scope of the CAN model beyond the focus on latent constructs of intra-attitudinal processes, and (2) studying network dynamics of health behaviour uptake at different points in time within the CAN model. First, the extension of the CAN model showed that by broadening the scope, we can increase the level of detail of the produced paths without hampering feasibility and make more specific recommendations regarding behaviour change as a result. In addition, we found that some constructs that are well-known for their predictive role in psychosocial models only played a moderate to minor role in determining our outcome behaviours, underscoring the added value of the broader scope. For example, perceived efficacy of PrEP played a moderate role in determining PrEP uptake, but only at earlier time points when PrEP use was probably less well integrated and knowledge in the community was limited. Likewise, in the network HIV risk perception was only peripherally connected to both of our target behaviours, suggesting that perceived exposure to risk was not central in BmPS decision-making.

Second, we demonstrated that the extended CAN model can be used to study changes in the structural importance of nodes. In the present study, structural importance of nodes rapidly changed across time points, while the connectivity of the networks remained stable. The latter is in contrast to previous theoretical work in which it is argued that connectivity increases over time once individuals become more familiar with (and think about) an attitude object^43^, thus increasing attitude strength. Accordingly, one could have expected a similar effect to occur here with the growing familiarity with BmPS. However, connectivity in our study was relatively low (with relatively high average shortest path lengths of 16.85–17.92) compared to other studies^41,43^ and did not increase. The reason that the connectivity remained relatively low could be that the attitudes are volatile as a result of changes in the availability and acceptability of strategies. This may provide inroads for behavioural change interventions since networks low in connectivity are easier to modify^41^. Studying temporal dynamics of networks may have the potential to provide insight into where interventions should be targeted at, at different stages of implementation, until a steady state of the network is reached, and the attitudes become more difficult to change.

Limitations of our approach relate predominantly to the inherent boundaries of the networks we presented. A network analysis like ours has as its goal to provide a comprehensive picture of the target behaviours, while at the same time, sufficient power is limited to networks of about 30 nodes per 500 participants^66^. Another limitation was the gap between T1 (July–December 2017) and T2 (July–December 2018) which limited us to specifically determine at which 6-monthly interval the significant change in network structure occurred. Finally, we acknowledge the temporal changes we found may be overestimated because of potential selection bias and low retest reliability and validity of the used measures. As for selection bias, we acknowledge the heterogeneity in our sample with only 41% of participants included at all time points Although we cannot rule this out, there were no significant differences in socio-demographics between time points, nor in socio-demographics or PrEP and VLS-related beliefs between those with (i.e., those included at all four time points) and without complete follow-up (data not shown), supporting the assumption of temporal changes rather than selection bias.

## Conclusion

Using an extension of the CAN model, we successfully identified several specific beliefs that related to the uptake of BmPS at different time points. We suggest that uptake may improve by discussing the positive impact of BmPS use on one’s quality of sex life and by generating more BmPS supportive social norms in the community. In addition, we recommend adjusting interventions to the stage of their implementation as factors that are associated with the uptake of BmPS correspond with changes in the accessibility, affordability and acceptability over time. We recommend future research to examine the value of our current approach including temporal dynamics in understanding and enhancing other health behaviours.

## Methods

### Study design and participants

We included HIV-negative participants from the Amsterdam Cohort Study (ACS) on HIV, which is an open prospective cohort of HIV-positive and HIV-negative MSM. The ACS was initiated in 1984 to investigate the epidemiology, pathogenesis and (natural) course of HIV, sexually transmitted infections (STIs) and blood-borne infections, and to evaluate the effect of interventions^16,67,68^. More details on the procedures of the study have been described earlier^67^. In brief: men are eligible for enrolment in the ACS if they are at least 18 years old, live in the Amsterdam area or are involved in MSM-related activities taking place in Amsterdam, and had sex with other men in the past 6 months. Every 6 months, participants visit the Public Health Service Amsterdam, the Netherlands, for HIV and STI testing, and complete a self-administered questionnaire on, among others, sexual behavior, applied HIV prevention strategies, and related psychosocial factors. For the current analysis, we included all MSM who visited the ACS between July 2017 and December 2019 and included per wave those MSM who were HIV-negative, reported anal sex in the past 6 months and completed a questionnaire (Time point 1: July–December 2017, Time point 2: July–December 2018, Time point 3: January–June 2019, Time point 4: July–December 2019). We excluded the data wave January–June 2018 as the questionnaire on VLS- and PrEP-related beliefs was not administered during that wave. We defined baseline as the first visit since 1 July 2017.

### Measures

#### Socio-demographic characteristics, sexual behavior, and use of HIV prevention strategies

We used data on socio-demographic characteristics (i.e., age, country of birth [Netherlands or other], education level [no college degree or at least college degree], residence area [in or outside Amsterdam]), sexual orientation (not exclusively homosexual or exclusively homosexual), having a steady partner (yes or no) and number of casual sex partners (0–5 or > 5 casual partners) in the past 6 months to describe the study population. At each of the time points, we measured condom use, PrEP use and VLS in the past 6 months.

#### Biomedical HIV prevention strategies and related variables included in the network

We included PrEP use and VLS in the past 6 months, socio-demographics, HIV risk perception, and a 21 PrEP-related and 9 VLS-related beliefs in the network analyses. Table 5 presents all the variables we included in the network and their short codes, the related items and their scale. The beliefs were informed by a previous study assessing PrEP use intention in the ACS^16^ relying on psychosocial theories such as TPB^34^ and the HBM^33^. The PrEP and VLS beliefs include injunctive and descriptive social norms, perceived self-efficacy, knowledge, perceived benefits and barriers of the use of BmPS (see Table 5).

**Table 5:** List of items and short codes included in the network, including the question and scale used in the questionnaire.

Item	Short code	Question	Scale
Use of biomedical HIV prevention			
PrEP use	PrEP	Did you use PrEP in the past 6 months?	0 = No; 1 = Yes
Viral load sorting	VLS	Did you use information about someone’s viral load to decide to have anal sex (without a condom) with that partner?	0 = No; 1 = Yes
Socio-demographics			
Age	Age	What is your date of birth?	Numeric field
Steady partner	SP	Did you have a steady partner in the past 6 months?	0 = No; 1 = Yes
HIV risk perception			
HIV risk perception	riskHIV	In the coming 6 months… …how worried are you about being infected with HIV? …what is the chance that you will be infected with HIV? …how severe would it be for you if you would be infected with HIV? …how important is it for you to prevent being infected with HIV?	1 (not worried)–7 (very worried) 1 (impossible)–7 (very likely) 1 (not severe)–7 (very severe) 1 (not important)–7 (very important)
PrEP beliefs			
Impact quality of sex life	prep1	PrEP use will improve the quality of my sex life	1 (totally disagree)–7 (totally agree)
Impact on serodiscordant couples	prep2	PrEP use will make it easier to engage in a sexual relationship with a potential HI- positive partner	1 (totally disagree)–7 (totally agree)
Solidarity towards HIV-positive individuals	prep3	PrEP use provides solidarity towards a potential HIV-positive partner	1 (totally disagree)–7 (totally agree)
Efficacy	prep4	PrEP use is effective enough to prevent risk for HIV	1 (totally disagree)–7 (totally agree)
Essential for high-risk	prep5	PrEP use is essential for individuals at high risk for HIV	1 (totally disagree)–7 (totally agree)
Redundant	prep6	PrEP use is redundant as there are other effective HIV prevention strategies available (e.g., condoms)	1 (totally agree)–7 (totally disagree)
Affordability	prep7	PrEP use is too expensive	1 (totally agree)–7 (totally disagree)
Expectation HIV drug resistance	prep8	PrEP use will increase the chance to develop HIV drug resistance	1 (totally agree)–7 (totally disagree)
Burden side-effects	prep9	PrEP use has many burdensome side-effects	1 (totally agree)–7 (totally disagree)
Burden PrEP procedures	prep10	PrEP use is accompanied with many unnecessary procedures	1 (totally agree)–7 (totally disagree)
Impact on sex life	prep11	PrEP use will negatively influence my sex life	1 (totally agree)–7 (totally disagree)
Opinion relevant others PrEP use for HIV prevention	prep12	People whose opinion I value (e.g., friends, partners, family members) view PrEP use as a good method to prevent HIV	1 (totally disagree)–7 (totally agree)
Gay friends use PrEP	prep13	Most of my gay friends uses PrEP as an HIV prevention strategy	1 (totally disagree)–7 (totally agree)
Opinion gay friends PrEP use	prep14	What will most of your gay friends think if you would use PrEP in the coming 6 months?	1 (very bad)–7 (very good)
PrEP associated with high-risk	prep15	In general, someone who uses PrEP is viewed as someone who takes more sexual risks	1 (totally agree)–7 (totally disagree)
PrEP associated with sexual health	prep16	In general, someone who uses PrEP is viewed as someone who takes responsibility for their sexual health	1 (totally disagree)–7 (totally agree)
PrEP associated with better sex life	prep17	In general, someone who uses PrEP is viewed as someone who has a better sex life	1 (totally disagree)–7 (totally agree)
PrEP associated with promsicuity	prep18	In general, someone who uses PrEP is viewed as someone who is promiscuous	1 (totally agree)–7 (totally disagree)
Easier to use than condoms	prep19	PrEP is more difficult to use than condoms	1 (totally agree)–7 (totally disagree)
Self-efficacy daily PrEP	prep20	How easy or difficult do you think it is… … to use PrEP daily? … to gain PrEP for daily use?	1 (very difficult)–7 (very easy)
Self-efficacy event-driven PrEP	prep21	How easy or difficult do you think it is… … to use PrEP on an event-driven basis? … to gain PrEP for event-driven use?	1 (very difficult)–7 (very easy)
Viral load sorting beliefs			
Prevents HIV transmission	vls1	Someone with HIV who has an undectable viral load can transmit an HIV infection through anal sex without a condom	1 (totally agree)–7 (totally disagree)
Protects serodiscordant couples	vls2	Viral load sorting protects of whom one is HIV-positive and one is HIV-negative	1 (totally disagree)–7 (totally agree)
Efficacy	vls3	Viral load sorting is effective enough to minimize my risk to be infected with HIV when I have sex without a condom	1 (totally disagree)–7 (totally agree)
Easier to use than condoms	vls4	Viral load sorting is easier to apply than using a condom	1 (totally disagree)–7 (totally agree)
Impact quality sex life	vls5	Applying viral load sorting will improve the quality of my sex life	1 (totally disagree)–7 (totally agree)
Opinion others to use viral load sorting for HIV prevention	vls6	People whose opinion I value (e.g., friends, partners, family members) view PrEP use as a good method to prevent HIV	1 (totally disagree)–7 (totally agree)
Gay friends use viral load sorting	vls7	Most of my gay friends uses viral load sorting as an HIV prevention strategy during anal sex	1 (totally disagree)–7 (totally agree)
Opinion gay friends application of viral load sorting	vls8	What will most of your gay friends think if you would use viral load sorting during anal sex in the coming 6 ?	1 (very bad)–7 (very good)
Self-efficacy	vls9	…Viral load sorting is easy to use as HIV prevention strategy …I am capable to use viral load sorting as HIV prevention strategy	1 (totally disagree)–7 (totally agree)

*PrEP* pre-exposure prophylaxis, *HIV* human immunodeficiency virus.

### Data analyses

First, all variables that measured the same belief and were measured with more than one item in the questionnaire (i.e., HIV risk perception and self-efficacy, see Table 5) were combined if their internal consistency exceeded 0.7 (Pearson’s correlation for 2 items or Cronbach’s alpha for > 2 items)^69^. The mean scores of the combined items were used in all further analyses.

Second, to examine the effect of time, we modelled the use of HIV prevention strategies (condoms, PrEP and VLS) and each PrEP and VLS related belief in logistic (for the HIV prevention strategies) and linear (for the beliefs) regression models using generalized estimating equations (GEE) to account for repeated measurements within individuals. In all models, we included the 6 monthly data waves as the time variable and the participant’s unique study id number to account for clustering.

Third, we estimated a weighted, undirected network for each time point, where we included pairwise interactions of PrEP and VLS use, age, having a steady partner, HIV risk perception, and 21 PrEP- and 9 VLS-related predictor variables (Table 5). To estimate the networks, we build on the work of Dalege et al.^39^. The networks were estimated using mixed graphical models, which allows inclusion of both binary and continuous data in contrast to other network estimation methods such as the eLasso procedure for binary^66^ or the Gaussian Graphical Model for continuous data^70^. The estimated networks resulted in sparse networks that represent each included variable as a node and display edges between the nodes that represent undirected, conditional dependent associations that are controlled for all other associations between nodes in the network. Edges between the continuous variables can be interpreted as partial correlations ranging from − 1 to 1, whereas for the binary nodes (PrEP, VLS and sp), a positive relation indicates that increasing the node results in a higher probability of outcome 1 of the factor (PrEP = 1: used PrEP in past 6 months, VLS = 1: VLS in past 6 months; sp = 1: reported a steady partner in the past 6 months). Using these estimated networks, we report on (1) *direct correlates* of PrEP and VLS use (using the edge weights between nodes and their bootstrapped 95% confidence intervals); (2) *community detection* to identify the clustering of beliefs closely related to each other (using the cluster walktrap algorithm [integrated in the *igraph* package] iterated 1000 times to select communities with nodes that belonged to the same community in over 90% of iterations); (3) *node centrality* to identify the nodes most influential in the networks (using node centrality measures); and (4) *temporal differences* between networks (using the Network Comparison Test). Popular indices of node centrality within a network are strength, betweenness and closeness^71^. In this paper, we only focused on strength as this indicator is most likely to produce stable parameter estimates^72^. Strength indicates the influence of a node on the network as it is based on the sum of the absolute edge values that are connected to a node. Influencing this node is most likely to impact the network as a whole^39^. We examined temporal differences with the network comparison test (NCT). This test assesses three things: first, whether the networks at each time point differ in network connectivity by testing differences in the global *strength* of a network (i.e., the sum of the absolute values of all edges in the network). Second, it determines whether the network *structure* is similar between time points by testing the maximum differences in edge weights between nodes of the networks, and, third, by testing which individual edge weights significantly differ across time points^73^. In addition, we have run two sensitivity analyses in which we estimated time trends in PrEP use and VLS use as outcomes and all other variables that were included in the network analyses in two logistic regression models using generalized estimating equations (GEE) to account for clustering within individuals. The results showed similar direct associations and effect sizes with PrEP and VLS as outcomes as the network analysis did (data not shown).

In our analyses we concentrated on choices of BmPS regardless of condom use because both condom users and non-condom users can choose to apply BmPS. We however conducted a range of sensitivity analyses to examine the change in the network structure if restricted to CAS, because behavioural factors such as absence of or inconsistent use of condoms could underlie the need to use BmPS, and, inconsistent condom use is a requirement to be enrolled in PrEP programs in the Netherlands. In these sensitivity analyses, we restricted the network analysis at the most recent time point to participants who reported CAS with casual partners in the past 6 months (N = 200 at T4) and tested for network invariance with the NCT. Additionally, we checked whether the baseline characteristics and associations of predictor variables with PrEP and VLS uptake were similar among this subgroup of the total sample (N = 258) compared to the total study population.

All network analyses were conducted in R version 3.6.3^74^. We used the R-package *mgm*^75^ to estimate the networks, *igraph*^76^ for community detection, *NetworkComparisonTest*^73^ to compare the networks across time points, and *qgraph*^77^ to visualize all networks. In addition, to estimate the accuracy of our networks at each time point, we used *bootnet* to examine the accuracy of edge weights and the centrality measure strength and assessed whether the estimated edges and strength of each edge significantly differed from each other^78^. All other statistical analyses were conducted in Stata version 15.1 ^79^. Results were considered significant at a *p*-value < 0.05.

### Ethical considerations

The ACS was approved by the Medical Ethics Committee of the Amsterdam University Medical Center of the University of Amsterdam, the Netherlands (MEC 07/182). Participation is voluntary and without incentive. Written informed consent of each participant was obtained before enrollment. All research was carried out in accordance with the Declaration of Helsinki.

### Supplementary Information

Supplementary Information.

## Data Availability

The anonymized complete ACS dataset will soon be made publicly available at https://www.amsterdamcohortstudies.org/acsc/menu/publicdata.asp. In the meantime, the data used for the current study can be requested through datamanagersoz@ggd.amsterdam.nl.
