# Supplementary material for: A psychosocial network approach studying biomedical HIV prevention uptake between 2017 and 2019
Source: Sci Rep. 2023 Sep 27;13:16168. doi: 10.1038/s41598-023-42762-2 (PMC10533833; doi:10.1038/s41598-023-42762-2)
Supplement: Supplementary file 1 — Supplementary Information. [file 41598_2023_42762_MOESM1_ESM.docx]

# A psychological network approach studying biomedical HIV prevention uptake over time

Authors

Hanne Zimmermann, Udi Davidovich, Ward van Bilsen, Liza Coyer, Amy Matser, Maria Prins, Frenk van Harreveld

# SUPPLEMENTARY MATERIALS

[S1. Edge weights at T1, T2, T3, T4 and T4 subsample CAS 2](#_Toc144912723)

[S2. Edge accuracy at T1, T2, T3 and T4 2](#_Toc144912724)

[S3. Edge differences at T1, T2, T3 and T4 2](#_Toc144912725)

[S4. Community detection at T1, T2, T3, and T4 2](#_Toc144912726)

[S5. Centrality accuracy at T1, T2, T3 and T4 3](#_Toc144912727)

[S6. Centrality differences at T1, T2, T3 and T4 3](#_Toc144912728)

[S7. Associations with PrEP uptake 4](#_Toc144912729)

[S8. Associations with VLS uptake 5](#_Toc144912730)

[S9. Comparison of demographic characteristics of the total sample and subsamples reporting or not reporting CAS 6](#_Toc144912731)

[S10. Comparison of the network at T4 among total sample and subsample reporting CAS 6](#_Toc144912732)

# S1. Edge weights at T1, T2, T3, T4 and T4 subsample CAS

See ‘S1-EdgeWeightsExcel_T1’ for the edge weights of the total sample at T1.

See ‘S1-EdgeWeightsExcel_T2’ for the edge weights of the total sample at T2.

See ‘S1-EdgeWeightsExcel_T3’ for the edge weights of the total sample at T3.

See ‘S1-EdgeWeightsExcel_T4’ for the edge weights of the total sample at T4.

See ‘S1-EdgeWeightsExcel_T4cas’ for the edge weights of the subsample reporting CAS at T4.

All available as excel files on OSF: <https://osf.io/qe62h/>

# S2. Edge accuracy at T1, T2, T3 and T4

See ‘S2-Edge accuracy_T1’ for the edge accuracy of the total sample at T1.

See ‘S2-Edge accuracy_T2’ for the edge accuracy of the total sample at T2.

See ‘S2-Edge accuracy_T3’ for the edge accuracy of the total sample at T3.
See ‘S2-Edge accuracy_T4’ for the edge accuracy of the total sample at T4.

All available as PDF files on OSF: <https://osf.io/qe62h/>

*Interpretation guidance: Bootstrapped confidence intervals of estimated edge-weights for the estimated network of BmPS uptake. The red line indicates the sample values and the gray area the bootstrapped CIs. Each horizontal line represents one edge of the network, ordered from the edge with the highest edge-weight to the edge with the lowest edge-weight. In the case of ties (for instance, multiple edge-weights were estimated to be exactly 0), the mean of the bootstrap samples was used in ordering the edges. Please note that negative edge weights can appear as positive edge weights in the edge accuracy output. This is because the edge weights are based on networks estimated with mgm in which the sign of the edge weights is stored separately, whereas the edge accuracy analysis is conducted with the bootnet that does not include the sign from mgm.*

# S3. Edge differences at T1, T2, T3 and T4

See ‘S3-Edge difference_T1’ for the edge differences of the total sample at T1.

See ‘S3-Edge difference_T2’ for the edge differences of the total sample at T2.

See ‘S3-Edge difference_T3’ for the edge differences of the total sample at T3.

See ‘S3-Edge difference_T4’ for the edge differences of the total sample at T4.

All available as PDF files on OSF: <https://osf.io/qe62h/>

*Interpretation guidance: Bootstrapped difference tests (α = 0.05) between edge-weights that were non-zero in the estimated network (above). Gray boxes indicate nodes or edges that do not differ significantly from one-another and black boxes indicate significant differences between edge weights. Colored boxes in the edge-weight plot correspond to the color of the edge in Figure 3.*

# S4. Community detection at T1, T2, T3, and T4

See ‘S4-Communitystability-T1’ for the community detection of the total sample at T1.

See ‘S4-Communitystability-T2’ for the community detection of the total sample at T2.

See ‘S4-Communitystability-T3’ for the community detection of the total sample at T3.

See ‘S4-Communitystability-T4’ for the community detection of the total sample at T4.

All available as PDF files on OSF: <https://osf.io/qe62h/>

*To identify highly interrelated factors in our networks (i.e. communities), we used the cluster walktrap algorithm (integrated in the igraph package) iterated 1000 times to select communities with nodes that belonged to the same community in over 90 percent of iterations. The PDF files of S4 present the output.*

# S5. Centrality accuracy at T1, T2, T3 and T4

See ‘S5a-Centrality accuracy_T1’ for the centrality accuracy of the total sample at T1.

See ‘S5a-Centrality accuracy_T2’ for the centrality accuracy of the total sample at T2.

See ‘S5a-Centrality accuracy _T3’ for the centrality accuracy of the total sample at T3.

See ‘S5a-Centrality accuracy_T4’ for the centrality accuracy of the total sample at T4.

See ‘S5b-Centrality accuracy_T1_order’ for the centrality accuracy of each node in the total sample at T1.

See ‘S5b-Centrality accuracy_T2_order’ for the centrality accuracy of each node in the total sample at T2.

See ‘S5b-Centrality accuracy_T3_order’ for the centrality accuracy of each node in the total sample at T3.

See ‘S5b-Centrality accuracy_T4_order’ for the centrality accuracy of each node in the total sample at T4.

All available as PDF files on OSF: <https://osf.io/qe62h/>

*Interpretation guidance: S5a figures show the stability of the strength measure. Here, the stability is quantified using the correlation stability coefficient, which quantifies the maximum proportion of cases that can be dropped to retain, with 95 % certainty, a correlation with the original centrality of higher than 0.7. For all time points, the correlation with the original sample is retained if cases are dropped.
S5b figures show the stability of the centrality measure strength for each node at each time point.*

# S6. Centrality differences at T1, T2, T3 and T4

See ‘S6-Centrality difference_T1’ for the centrality differences of the total sample at T1.

See ‘S6-Centrality difference_T2’ for the centrality differences of the total sample at T2.

See ‘S6-Centrality difference_T3’ for the centrality differences of the total sample at T3.

See ‘S6-Centrality difference_T4’ for the centrality differences of the total sample at T4.

All available as PDF files on OSF: <https://osf.io/qe62h/>

*Interpretation guidance: Bootstrapped difference tests (α = 0.05) between node strength of the nodes (below). Black boxes indicate significant differences in centrality between nodes with α=.05, node strength is presented in the diagonal boxes.*

# S7. Associations with PrEP uptake

| **S7. Multivariable logistic GEE models of socio-demographic and beliefs about biomedical HIV prevention strategies and their association with PrEP use over time among men who have sex with men within the Amsterdam Cohort Studies, July 2017 -December 2019, Amsterdam, the Netherlands.** | | | | | | |
| --- | --- | --- | --- | --- | --- | --- |
|  | **Total sample  (N=632)** | | | **Participant reporting CAS (N=258)** | | |
|  | **Multivariable GEE model** | | | **Multivariable GEE model** | | |
|  | **aOR** | **95%CI** | ***p*-value** | **aOR** | **95%CI** | ***p*-value** |
| Time | 1.24 | 1.09-1.41 | 0.001 | 1.30 | 1.15-1.47 | <0.001 |
| Age | 1.03 | 1.01-1.05 | 0.003 | 1.02 | 1.01-1.05 | 0.008 |
| Having a steady partner |  |  |  | 1.99 | 1.31-3.05 | 0.001 |
| HIV risk perception | 0.74 | 0.61-0.90 | 0.003 | 0.50 | 0.37-0.67 | <0.001 |
| Applying viral load sorting | 1.84 | 1.15-2.95 | 0.011 |  |  |  |
| **PrEP beliefs** |  |  |  |  |  |  |
| Impact quality of sex life | 1.20 | 1.06-1.36 | 0.003 | 1.18 | 1.02-1.38 | 0.028 |
| Impact on serodiscordant couples |  |  |  |  |  |  |
| Solidarity towards HIV-positive individuals |  |  |  |  |  |  |
| Efficacy |  |  |  |  |  |  |
| Essential for high-risk |  |  |  |  |  |  |
| Redundant |  |  |  |  |  |  |
| Affordability |  |  |  |  |  |  |
| Resistance development HIV medication | 0.86 | 0.76-0.96 | 0.008 | 0.78 | 0.67-0.90 | 0.001 |
| Burden side-effects | 1.42 | 1.23-1.65 | <0.001 | 1.47 | 1.25-1.73 | <0.001 |
| Burden PrEP procedures |  |  |  |  |  |  |
| Impact on sex life | 0.81 | 0.70-0.94 | 0.006 |  |  |  |
| Opinion relevant others PrEP use for HIV prevention |  |  |  |  |  |  |
| Gay friends use PrEP | 1.31 | 1.19-1.44 | <0.001 | 1.24 | 1.10-1.40 | <0.001 |
| Opinion gay friends PrEP use | 1.21 | 1.05-1.39 | 0.007 |  |  |  |
| Association increased sexual risk taking |  |  |  |  |  |  |
| Association sexual health responsibility |  |  |  | 1.18 | 1.00-1.39 | 0.046 |
| Association better sex life |  |  |  | 1.16 | 1.00-1.36 | 0.046 |
| Association promiscuity |  |  |  |  |  |  |
| Easier to use than condoms |  |  |  |  |  |  |
| Self-efficacy daily PrEP | 1.39 | 1.20-1.61 | <0.001 | 1.43 | 1.21-1.70 | <0.001 |
| Self-efficacy event-driven PrEP |  |  |  |  |  |  |
| **Viral load sorting beliefs** |  |  |  |  |  |  |
| Prevents HIV transmission |  |  |  |  |  |  |
| Protects serodiscordant couples |  |  |  |  |  |  |
| Efficacy |  |  |  |  |  |  |
| Easier to use than condoms |  |  |  |  |  |  |
| Impact quality sex life |  |  |  |  |  |  |
| Opinion others to use viral load sorting for HIV prevention |  |  |  |  |  |  |
| Gay friends use viral load sorting |  |  |  |  |  |  |
| Opinion gay friends application of viral load sorting |  |  |  |  |  |  |
| Self-efficacy |  |  |  |  |  |  |

Abbreviations: GEE = generalized estimated equation; PrEP = pre-exposure prophylaxis; HIV = Human Immunodeficiency Virus; CAS = condomless anal sex.

# S8. Associations with VLS uptake

| **S8. Multivariable logistic GEE models of socio-demographic and beliefs about biomedical HIV prevention strategies and their association with viral load sorting over time among men who have sex with men within the Amsterdam Cohort Studies, July 2017 -December 2019, Amsterdam, the Netherlands.** | | | | | | |
| --- | --- | --- | --- | --- | --- | --- |
|  | **Total sample  (N=632)** | | | **Participant reporting CAS (N=258)** | | |
|  | **Multivariable GEE model** | | | **Multivariable GEE model** | | |
|  | **aOR** | **95%-CI** | ***p*-value** | **aOR** | **95%-CI** | ***p*-value** |
| Time |  |  |  |  |  |  |
| Age |  |  |  |  |  |  |
| Having a steady partner |  |  |  |  |  |  |
| HIV risk perception | 1.38 | 1.14-1.65 | 0.001 |  |  |  |
| PrEP use | 2.17 | 1.29-3.68 | 0.004 |  |  |  |
| **PrEP beliefs** |  |  |  |  |  |  |
| Impact quality of sex life |  |  |  |  |  |  |
| Impact on serodiscordant couples |  |  |  |  |  |  |
| Solidarity towards HIV-positive individuals |  |  |  |  |  |  |
| Efficacy |  |  |  |  |  |  |
| Essential for high-risk |  |  |  |  |  |  |
| Redundant |  |  |  |  |  |  |
| Affordability |  |  |  |  |  |  |
| Resistance development HIV medication |  |  |  | 0.82 | 0.69-0.99 | 0.039 |
| Burden side-effects |  |  |  |  |  |  |
| Burden PrEP procedures |  |  |  |  |  |  |
| Impact on sex life |  |  |  |  |  |  |
| Opinion relevant others PrEP use for HIV prevention |  |  |  |  |  |  |
| Gay friends use PrEP |  |  |  |  |  |  |
| Opinion gay friends PrEP use |  |  |  | 1.27 | 1.00-1.60 | 0.048 |
| Association increased sexual risk taking | 1.23 | 1.08-1.42 | 0.002 | 1.17 | 1.00-1.36 | 0.049 |
| Association sexual health responsibility |  |  |  |  |  |  |
| Association better sex life |  |  |  |  |  |  |
| Association promiscuity |  |  |  |  |  |  |
| Easier to use than condoms |  |  |  |  |  |  |
| Self-efficacy daily PrEP |  |  |  |  |  |  |
| Self-efficacy event-driven PrEP |  |  |  |  |  |  |
| **Viral load sorting beliefs** |  |  |  |  |  |  |
| Prevents HIV transmission |  |  |  |  |  |  |
| Protects serodiscordant couples |  |  |  |  |  |  |
| Efficacy | 1.16 | 1.02-1.32 | 0.029 |  |  |  |
| Easier to use than condoms |  |  |  |  |  |  |
| Impact quality sex life |  |  |  |  |  |  |
| Opinion others to use viral load sorting for HIV prevention | 0.83 | 0.70-0.98 | 0.o30 | 0.75 | 0.61-0.92 | 0.005 |
| Gay friends use viral load sorting | 1.19 | 1.02-1.37 | 0.023 | 1.24 | 1.03-1.50 | 0.022 |
| Opinion gay friends application of viral load sorting |  |  |  |  |  |  |
| Self-efficacy | 1.29 | 1.06-1.57 | 0.012 | 1.30 | 1.04-1.61 | 0.018 |

Abbreviations: PrEP = pre-exposure prophylaxis; HIV = Human Immunodeficiency Virus; CAS = condomless anal sex.

# S9. Comparison of demographic characteristics of the total sample and subsamples reporting or not reporting CAS

| **S9. Demographic characteristics of HIV-negative MSM reporting or not reporting CAS of the Amsterdam Cohort Studies, July 2017-December 2019, Amsterdam, the Netherlands**. | | | | | |
| --- | --- | --- | --- | --- | --- |
|  | **Total sample  (N=632)** | **Participant reporting CAS**  **(N=258)** | **Particants not reporting CAS**  **(N=374)** | ***p*-value** |  |
| Age at baseline^a^ (median, IQR) | 41 (31-49) | 41 (30-49) | 42 (33-49) | 0.322 |  |
| Born in the Netherlands | 527 (83%) | 215 (83%) | 309 (83%) | 0.815 |  |
| Living in Amsterdam | 523 (83%) | 208 (80%) | 315 (84%) | 0.238 |  |
| Exclusively homosexual | 509 (81%) | 201 (80%) | 308 (82%) | 0.165 |  |
| College degree or higher | 504 (80%) | 204 (79%) | 300 (80%) | 0.725 |  |
| Having a steady partner at baseline^a^ | 408 (65%) | 116 (45%) | 292 (78%) | <0.001 |  |

Abbreviations: HIV= Human Immunodeficiency Virus; MSM = men who have sex with men; CAS = condomless anal sex.

^a^Baseline was defined as the first visit of the participant between 1 July 2017 and 31 December 2019.

# S10. Comparison of the network at T4 among total sample and subsample reporting CAS

See ‘S9-Sensitivity 49vs49cas_v4’ for the estimated network at T4 among the total sample and the subsample and the NCT figure comparing these networks.

All available as PDF files on OSF: <https://osf.io/qe62h/>

*Interpretation guidance****:*** *Estimated networks of PrEP use, VLS and related factors at the total study population at T4 compared to the subgroup reported not using condoms in the past 6 months at T4 (first two figure from left) and significant differences in edge weights between these network (right figure). For the interpretation of the networks, see Figure’s 3 caption. For the interpretation of the NCT: The magnitude of the edge differences is indicated by edge width. A blue edge indicates that the relation (based on edge weights) is significantly weaker, absent or more negative at T4 among the total study population compared to T4 among the subgroup reporting CAS. A red edge indicates that the relation is significantly weaker, absent or more negative at T4 among the total study population.*

*Please note that the NCT compares networks of either continuous or binary variables, and not from mixed networks using mgm. The results of the NCT (i.e. significant differences between edges) are therefore based on the networks estimated for continuous variables (with EBICglasso). The NCT graph displays those edges that differ significantly according to the results of the NCT with the displayed difference in strength based on the edge weight in the mgm network.*
